# An essential role for the VASt domain of the Arabidopsis VAD1 protein in the regulation of defense and cell death in response to pathogens

**DOI:** 10.1371/journal.pone.0179782

**Published:** 2017-07-06

**Authors:** Mehdi Khafif, Claudine Balagué, Carine Huard-Chauveau, Dominique Roby

**Affiliations:** LIPM, Université de Toulouse, INRA, CNRS, Castanet-Tolosan, France; University of Maryland Baltimore County, UNITED STATES

## Abstract

Several regulators of programmed cell death (PCD) have been identified in plants which encode proteins with putative lipid-binding domains. Among them, VAD1 (Vascular Associated Death) contains a novel protein domain called VASt (VAD1 analog StAR-related lipid transfer) still uncharacterized. The Arabidopsis mutant *vad1-1* has been shown to exhibit a lesion mimic phenotype with light-conditional appearance of propagative hypersensitive response-like lesions along the vascular system, associated with defense gene expression and increased resistance to *Pseudomonas* strains. To test the potential of ectopic expression of *VAD1* to influence HR cell death and to elucidate the role of the VASt domain in this function, we performed a structure-function analysis of VAD1 by transient over-expression in *Nicotiana benthamiana* and by complementation of the mutant *vad1-1*. We found that (i) overexpression of *VAD1* controls negatively the HR cell death and defense expression either transiently in *Nicotiana benthamania* or in Arabidopsis plants in response to avirulent strains of *Pseudomonas syringae*, (ii) VAD1 is expressed in multiple subcellular compartments, including the nucleus, and (iii) while the GRAM domain does not modify neither the subcellular localization of VAD1 nor its immunorepressor activity, the domain VASt plays an essential role in both processes. In conclusion, VAD1 acts as a negative regulator of cell death associated with the plant immune response and the VASt domain of this unknown protein plays an essential role in this function, opening the way for the functional analysis of VASt-containing proteins and the characterization of novel mechanisms regulating PCD.

## Introduction

Programmed cell death (PCD) occurs in plants in a variety of cell types during development and to face environmental stresses such as pathogens or other stress signals [1, 2]. The Hypersensitive Response (HR) is one of the best characterized PCD, which is localized at the site of attempted pathogen invasion. In many plant-pathogen interactions, HR is triggered upon pathogen recognition. The plant immune system contains several layers [3]. The basal layer of defense, referred to as PAMP Triggered Immunity (PTI), is activated by the presence of pattern recognition receptors (PRRs) which recognize Microbe–or Pathogen-Associated Molecular Patterns (MAMPs or PAMPs). PRRs are plasma membrane receptors, that activate rapid and effective immune responses upon PAMP recognition [4]. However, pathogenic microorganisms can suppress PTI by producing effector proteins, thus leading to plant susceptibility to pathogen multiplication. However, a second and more robust layer of defense response, termed Effector Triggered Immunity (ETI) occurs upon recognition of pathogen effectors by a second type of plant immune receptors (R proteins). In many cases, ETI leads to the hypersensitive cell death, leading to the confinement of the pathogens at the attempted infection site.

PCD is highly regulated at the genetic level as the control of this cell death programme is essential for the development of multicellular organisms. In plants, its fundamental role in response to pathogens has been revealed by the fact that many pathogens have evolved different strategies to suppress the HR. The molecular mechanisms leading to cell death after pathogen attack during ETI are not fully elucidated. However, some regulators or executioners of HR have been identified, such as BI-1 (BAX INHIBITOR 1, [5, 6], caspases and metacaspases [7] or MYB30 [8]. Characterization of mutants displaying spontaneous HR-like cell death on leaves, the so-called lesion mimic mutants (LMMs) has also been frequently reported, leading to the identification of genes encoding inhibitors of plant cell death, frequently associated with immunity [9, 10]. Demonstration of their implication in immunity control has been made in several cases, either by effector targeting [11, 12], by genetic interaction with immunity pathway components [13, 14, 15] or by suppressor identification [10]. In summary, numerous autoimmune or LMMs and their suppressors represent genes (potentially) involved in the control of the HR, and should be characterized more in depth [16].

We previously reported that a LMM loss-of-function mutant of the gene *VASCULAR ASSOCIATED DEATH1* (*VAD1*) exhibits constitutive immune responses and HR-like spontaneous lesions [14]. The *vad1* phenotypes are suppressed by mutations affecting immunity pathways such as *eds1* and *npr1*, and by mutations affecting hormonal pathways (salicylic acid and ethylene pathways), known to control the immune response [17, 14]. In contrast to most *LMM* mutants, *vad1* is affected in the control of cell death propagation instead of its initiation [14]. *VAD1* encodes a protein of unknown function containing a GRAM domain predicted to bind lipids [18, 19] and a VASt (VAD1 Analog of StAR-related lipid transfer) domain. This domain which belongs to the Bet v1-like superfamily [5], has no molecular function assigned yet. Interestingly, among many uncharacterized proteins containing both a VASt and a GRAM domain, the yeast YSP2 (Yeast Suicide protein 2) gene encodes a cell death regulator [20]. These findings open new perspectives for the functional analysis of VAD1. It should be noted that a significant proportion of plant LMM mutants are affected in genes associated with lipid synthesis or homeostasis [10]. It is the case of *acd5* (*Accelerated Cell Death 5*) mutant which is deficient in a ceramide kinase [21], *erh1* (*Enhancing RPW8- mediated HR-like cell death*) which lacks the inositol-phosphorylceramide synthase 2 (IPCS2)[22], *acd11* (*Accelerated Cell Death 11*) which is affected in the ceramide-1-phosphate transfer protein (C1P)[23], or *loh1* (*LAG One Homolog*) affected in a ceramide synthase [24]. This reveals that the lipid metabolism plays a major role in promoting PCD. In this context, it is interesting to note that the *vad1* phenotypes are dependent on the PATATIN-LIKE PROTEIN 2 (PLP2), a pathogen induced patatin like lipid acyl hydrolase [25].

In this study, a functional analysis of VAD1 was performed. Our results show that (i) overexpression of *VAD1* controls negatively the HR cell death and defense expression either transiently in *Nicotiana benthamania* or in Arabidopsis plants in response to avirulent strains of *Pseudomonas syringae*, (ii) while the GRAM domain does not modify neither the subcellular localization of VAD1 nor its immunorepressor activity, the domain VASt plays an essential role in both processes.

## Materials and methods

### Plant material

As previously described, mutant and wild-type seeds were sterilized and sown on Murashige and Skoog plates [26], supplemented with the appropriate antibiotic under a light period of 16 h (71 μmol m^−2^ s^−1^) at 21°C. Arabidopsis mutant (*vad1-1*) and other transgenic lines used in this study (*35S*::*RFP*::*VAD1*, *35S*::*RFP*::*ΔGRAM*, *35S*::*RFP*::*ΔVASt*) are in the WS-4 or *vad1-1* background and were grown respectively in presence of kanamycin 50 μg mL^−1^ for the *vad1-1* mutant or hygromycin 25 μg mL^−1^ for the transgenic lines.

7 to 10 days after sowing, seedlings were transplanted to Jiffy pots and grown in a growth chamber under a light period of 9 h (192 μmol m^−2^ s^−1^) at 21°C and 40% to 70% relative humidity and4- to 5-week old plants were used for most experiments. Under these growth conditions, lesions appear on the *vad1-1* mutant 21 days after transplanting.

### Bacterial growth and plant infection

For transient expression of proteins in *N*. *benthamiana*, bacterial cultures of *Agrobacterium tumefaciens* (strain C58C1) were grown in YEB medium supplemented with the appropriate antibiotics: rifampicin 50 *μ*g mL^−1^, tetracyclin 50 *μ*g mL^−1^ and spectinomycin 50 *μ*g mL^−1^ for strains carrying the different VAD1 constructs. Bacterial cultures of *Agrobacterium tumefaciens* (strain GV3101) (MtSYMREM1) were grown in YEB medium supplemented with rifampicin 50 μg mL^−1^, carbenicillin 50 μg mL^−1^, and gentamycin 50 μg mL^−1^. After centrifugation, cells were resuspended in induction buffer (10 mM MgCl2, 10 mM Mes, pH 5.6, and 200 μM acetosyringone) to an OD_600_ of 0.5. Leaves of 4-week-old *N*. *benthamiana* grown at 22°C were inoculated and then placed in a growth chamber. Five hours after, the same leaves were inoculated with an *A*. *tumefaciens* strain carrying the avirulent gene (*AvrRpt2* or *AvrRpt0*). One or two days after *A*. *tumefaciens* infiltration, leaf discs were harvested and processed, or frozen immediately in liquid nitrogen and stored at –80°C.

Transient expression of proteins in Arabidopsis seedlings was performed as previously described [12]. Briefly, *A*. *tumefaciens* overnight cultures were collected and resuspended at an OD_600_ of 1 in 2 mL of Arabidopsis liquid medium containing 200 μM acetosyringone. Seedlings were vacuum infiltrated (10 mm Hg) twice for 1 min with the *A*. *tumefaciens* solution. Excess infiltration medium was subsequently removed and the plates transferred to a culture room (22°C, long day conditions, 150 μmol m^−2^ s^−1^ light intensity) for 3 days.

The avirulent *Pseudomonas syringae* pv. *tomato* strains were grown at 28°C on King's B medium supplemented with antibiotics (rifampicin 50 μg mL^−1^, kanamycin 50 μg mL^−1^ and tetracyclin 10 μg mL^−1^) (DC3000/AvrRpm1). Four- or 5-week-old plants were used for bacterial inoculation. After 12 h at high humidity, plants were grown under light-promoting lesions under the following conditions: 9h light/15h dark and 90% relative humidity [14]. Plants were infiltrated with a bacterial suspension of 5 × 10^6^ colony forming units (CFU) mL^−1^ (DC3000/avrRpm1) for visualization of HR phenotypes, 1 × 10^7^ CFU mL^−1^ for gene expression analysis and 2 × 10^7^ CFU mL^−1^ for runaway cell death experiments. Determination of *in planta* bacterial growth was performed as previously described [14].

### RNA extraction and quantitative real-time PCR (qRT-PCR)

Total RNA extraction was performed from leaves with the Nucleospin RNA kit (Macherey-Nagel). cDNA was synthesized with Superscript III reverse transcriptase (Invitrogen Carlsbad, CA) using 1 μg of total RNA. Quantitative RT-PCR was performed using gene-specific primers (S1 Table), on a Light Cycler 480 II apparatus (Roche Diagnostics, Meylan, France) using Roche reagents. SYBR green I. Five pmol of each primer and 1 μL of a five-fold dilution of reverse transcriptase reaction product were used in a final reaction volume of 10 μL with PCR cycling conditions as follows: 9 min at 95°C, followed by 45 cycles of 5 s at 95°C, 10 s at 65°C and 20 s at 72°C. At least three independent biological assays were used for Quantitative PCR reactions. All reactions were checked for their dissociation curves. ΔCT between each gene and the internal controls (*At5g16260*, for *Arabidopsis thaliana* experiments and *At5g15710* for *N*. *benthamiana* experiments [27] was then calculated for each sample, and expression levels for each transcript were calculated as 2–ΔCT and expressed in arbitrary units.

### Quantification of cell death using electrolyte leakage

Three leaf discs (6 mm diameter) were harvested 24 hours post-inoculation (hpi) with the indicated strains, washed, and incubated at room temperature in 10 mL of distilled water. Leaf discs were then transferred into 5mL of fresh distilled water and incubated 1 hour at room temperature before measuring conductivity. Two to five independent experiments were performed with six plants (three leaves per plant). For the runaway cell death monitoring in Arabidopsis lines, 3 leaf discs (0.4 mm diameter) were used and the leaf discs of 2 plants were pooled into one vial containing 3.5mL of fresh distilled water. Two independent experiments were performed with 9–15 plants.

### Protein extraction and western blotting

Total protein was extracted from leaves harvested 24h after treatment (transient expression experiments) or 2–3 weeks after sawing (stable expression experiments) using the Laemmli buffer [28] and separated on SDS-PAGE. For CFP tagged proteins, blots were incubated with rabbit anti-GFP (AMS Biotechnology, [1:5,000]) antibody and a goat anti-rabbit IgG HRP-coupled secondary antibody (Millipore [1:10,000]). For RFP-tagged proteins, blots were incubated with rabbit anti-RFP HRP-coupled antibody (1:5,000) goat anti-rabbit secondary antibody (Millipore [1:10,000]). Proteins were visualized using the Immobilon kit (Millipore).

### Gene cloning and Arabidopsis transformation

Unless otherwise indicated, plasmids used in this study were constructed using the Gateway technology (GW; Invitrogen) following the instructions of the manufacturer. To generate the VAD promoter::VAD1::GFP construct, the *VAD1* genomic sequence including 604 nucleotides upstream the initiation site was amplified using pfx DNA polymerase (Invitrogen) and the T7I23 BAC clone as DNA template. The PCR product was then digested with BamHI and NotI restriction enzymes to be cloned into the pENTR4 vector and then by a BP reaction into pMDC110 (Invitrogen). To generate the 35S::RFP::VAD1 constructs, the open reading frame (ORF) of *VAD1* was cloned into the binary vector, downstream of the CaMV 35S and the RFP tag. PCR products flanked by the attB sites were cloned into the pDONR207 vectors (Invitrogen) *via* a BP reaction to create the corresponding entry clones with attL sites. Inserts cloned into the entry clones were subsequently recombined into the destination vector pH7WGR2, obtained from Plant System Biology (Ghent University; http://gateway.psb.ugent.be/) *via* an LR reaction to create the expression constructs. All the cloned PCR products were confirmed by sequencing. Arabidopsis *vad1-1* lines were transformed with the different constructs by the floral dipping method [29]. T1 seedlings were screened on MS plate supplemented with hygromycin B 25 μg mL^−1^. Homozygous transformant lines were confirmed by segregation experiments using T3 seedlings. All the primers used for cloning are listed in S1 Table.

### Fluorescence microscopy

Fluorescence images were acquired using a Leica SP8 confocal microscope equipped with a water immersion objective lens (× 25, numerical aperture 1.20; PL APO). GFP fluorescence was excited with the 488 nm ray line of the argon laser and recorded in one of the confocal channels in the 505 to 530 nm emission range. RFP fluorescence was excited with the 561 nm ray line of the He-Ne laser and detected in the range between 595 and 620 nm. Images were acquired in the sequential mode using Leica LCS software and analyzed using the ImageJ software. A set of fluorescent protein fusion constructs that mark different sub-cellular compartments has been used, including *AtMYB30* (*At3g28910*, nucleus) [11], *AtERD2* (*At1g29330)*, Golgi-cis/ER network or *AtWAK2 fused to HDEL retention peptide*, ER) [30, 31], *γ-TIP* (tonoplast intrinsic protein) (*At2g36830*, tonoplast membrane) [16], *AtRANBP1A* (*At1g07140*, cytoplasm/nuclear export) [32] and *PMA4*, (*X66737*, plasma membrane) [33].

### Statistics

Each experiment was independently conducted 2–4 times with similar results. The results are shown as means ± standard deviation. For Q-RT-PCR, samples consisted of pools of leaf discs collected from 5 plants for bacterial internal growth quantification or 9–15 plants for cell death quantification. Data were analyzed by Student's t-test or Kruskal and Wallis one-way analysis of variance followed by post-hoc tests for pairwise multiple comparisons (Nemenyi's test with Tukey-Dist approximation for independent samples) or nonparametric multiple comparison procedures for unbalanced one-way factorial designs [34]. All these analyses were performed using the R statistical software system (http://www.cran.r-project.org, Nparcomp R package version 2.6, PMCMR R package).

## Results

### Transient expression of VAD1 leads to HR cell death and defense suppression

**Fig 1:**
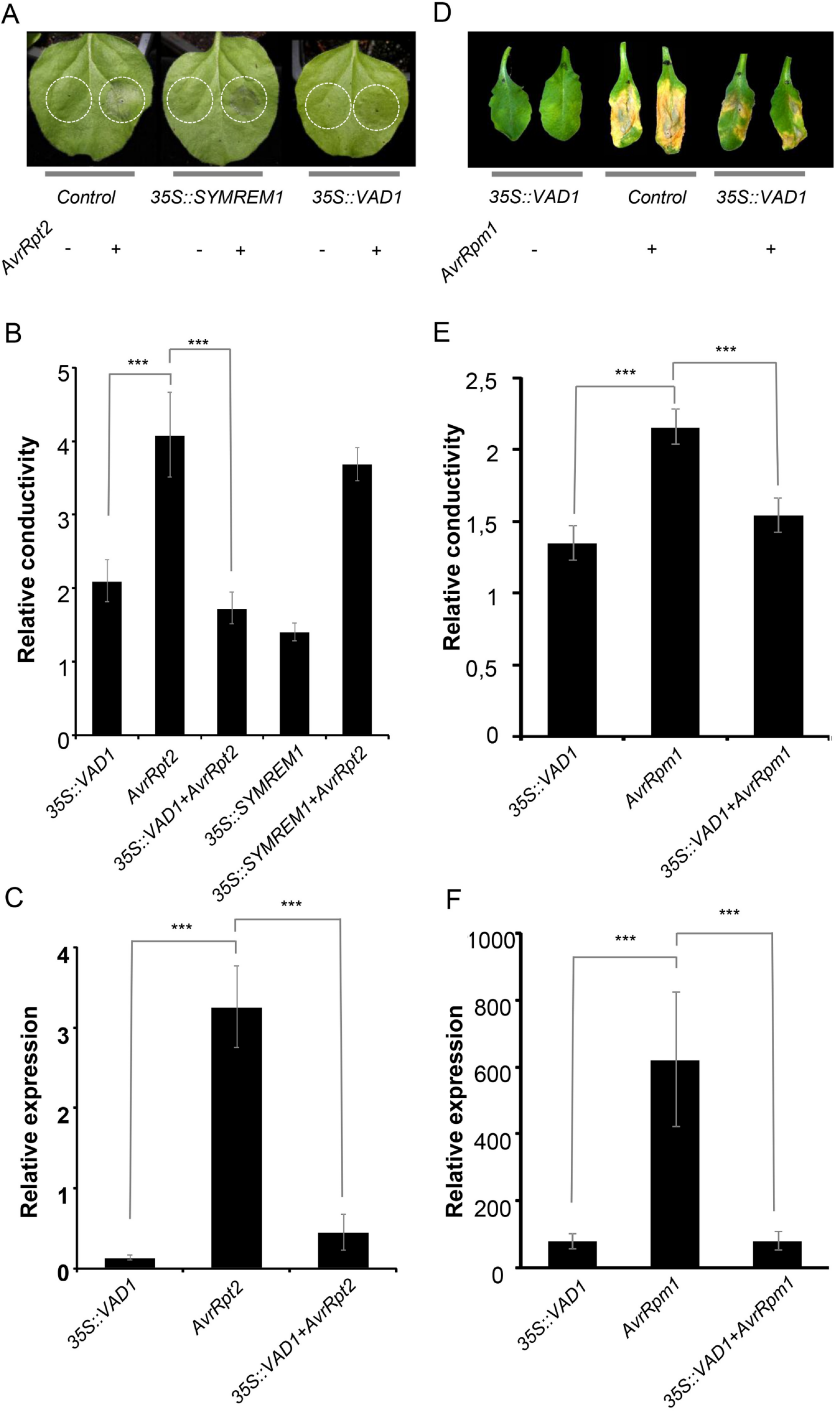
*VAD1* overexpression leads to suppression of HR cell death and defense in response to bacterial avirulence effectors or avirulent pathogens. (A) VAD1 delays and reduces HR induced by AvrRpt2 after agroinfiltration of *N*. *benthamiana* leaves. AvrRpt2 was agroinfiltrated on leaves (marked by white circles) to induce HR and observations were made 24 hours post-infiltration. Except for the control (AvrRpt2), all areas were co-agroinfiltrated with *35S*::*RFP*::*VAD1*, or *35S*::*SYMREM1*::*CFP* (a control protein). (B) Quantification of cell death by measuring electrolyte leakage 24 h after agroinfiltration of *N*. *benthamiana* leaves with the indicated strains (OD 0.5 for constructs, 0.3 for AvrRpt2). Data are expressed relative to AvrRpt2 data. Statistically significant differences were determined using t-test (* indicates P < 0.05). (C) Expression analysis of the *PR1a* defense gene in *N*. *benthamiana* leaves after agroinfiltration with the indicated strains (OD 0.5 for constructs and OD 0.3 for AvrRpt2). Mean and SE values were calculated from the results of a representative out of 3 independent experiments with 3 individual plants (four leaves/plant). Statistically significant differences were determined using t-test (* indicates P < 0.05). (D) VAD1 delays and reduces HR induced by *Pst* DC3000 harboring *AvrRpm1*. The bacterial strain was inoculated on Arabidopsis leaves to induce HR (indicated by the tissue collapse at the inoculation site). Leaves were shown 30 hours post-inoculation with DC3000 AvrRpm1. Except for the control (AvrRpm1), all areas were co-agroinfiltrated with *35S*::*RFP*::*VAD1*, or *35S*::*SYMREM1*::*CFP* (a control protein). (E) Quantification of cell death by measuring electrolyte leakage 48h after inoculation of leaves with *Pst* DC3000 AvrRpm1 (1x10^7^ CFU.mL^-1^). Data are expressed relative to AvrRpm1 data 9h after sampling. Statistically significant differences were determined using t-test (* indicates P < 0.05). (F) Expression analysis of the *PR1* defense gene in *A*. *thaliana* leaves after inoculation with *Pst* DC3000 AvrRpm1 (1x10^7^ CFU.mL^-1^). Mean and SE values were calculated from the results of a representative out of 3 independent experiments with 3 individual plants (four leaves/plant). Statistically significant differences were determined using t-test (* indicates P < 0.05).

We previously demonstrated that a loss of function mutation of *VAD1* was associated with appearance of propagative HR-like lesions along the vascular system and increased resistance to virulent and avirulent pathogens [14]. We also found that the *vad1* phenotypes were dependent on ETI signaling components such as NDR1 and EDS1 [14]. To determine whether VAD1 acts as a negative regulator of immunity and has a direct role in suppressing cell death, we analyzed its overexpression effect on PCD. First we tested the effect of overexpression of VAD1 by *Agrobacterium*-mediated transient expression of a *35S*::*RFP*::*VAD1* fusion in leaf epidermal cells of *Nicotiana benthamiana* (*N*. *benthamiana*) leaves, on the HR induced by the expression of the *Pseudomonas* effector AvrRpt2 [35]. As shown in Fig 1A, expression of *VAD1* has a negative effect on the development of the AvrRpt2-triggered HR, whereas the expression of another unrelated protein (MtSYMREM1, [36]) does not affect HR progress. These visual observations were confirmed by ion leakage measurements in leaf disk assays 24h after plant agro-infiltration with the different strains (Fig 1B). Interestingly, suppression of cell death correlated with decreased defense gene expression. Indeed, *PR1a* expression was drastically reduced when *VAD1* was co-expressed with AvrRpt2 (Fig 1C). We additionally tested the effect of *VAD1* overexpression on HR induced by another avirulence gene *AvrPto* [35] (S1 Fig), with similar results. Therefore, overexpression of *VAD1* in *N*. *benthamiana* greatly decreased HR induced by two different effector proteins.

We subsequently analyzed the effect of overexpression of VAD1 on PCD induced by *Pseudomonas syringae* pv. *tomato* (*Pst*) DC3000 carrying the avirulent effector AvrRpm1 in Arabidopsis [37]. At 12 hours post-inoculation, a strong HR was observed on all leaves inoculated with *Pst* DC3000 *AvrRpm1* alone, while HR was delayed and reduced when *35S*::*VAD1*::*GFP* was simultaneously agroinfiltrated (Fig 1D). These observations were confirmed by ion leakage measurements and *PR1* gene expression (Fig 1E and 1F).

Taken together, these data show that overexpression of *VAD1* in Arabidopsis and *N*. *benthamiana* greatly reduced HR induced respectively, either by two different avirulence effectors, or by an avirulent strain of *Pst*.

### VAD1 is localized in multiple subcellular compartments

**Fig 2:**
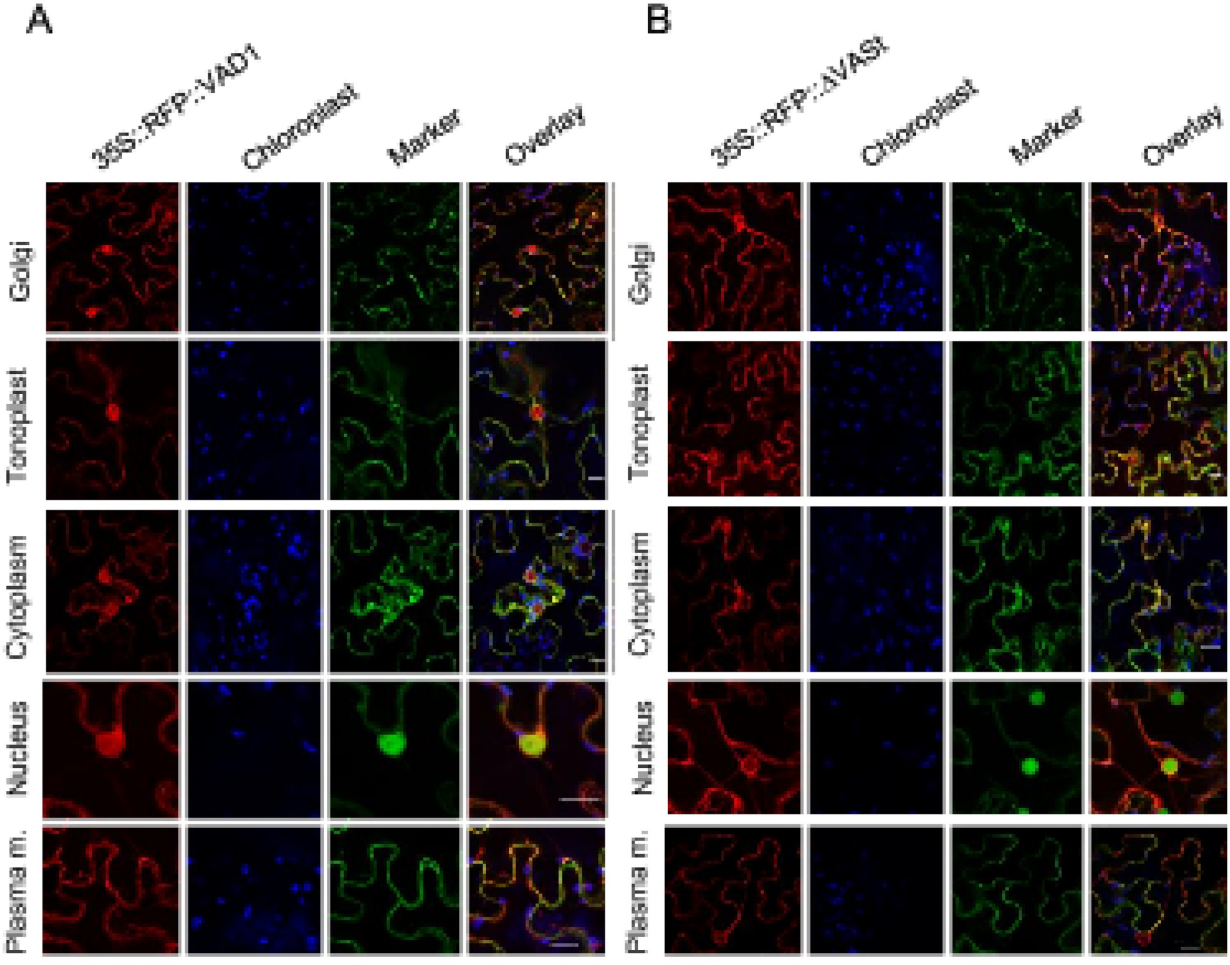
VAD1 localizes in multiple subcellular compartments and a VASt domain deletion excludes VAD1 from the nucleus. (A) Transient co-expression by agroinfiltration of *N*. *benthamiana* leaves of *35S*::*RFP*::*VAD1* construct (left panels), with subcellular markers (central panels, ERD2–GFP for the Golgi, γ-TIP–GFP for the tonoplast, GFP for the cytoplasm, MYB30-GFP for the nucleus, PMA4–GFP for the plasma membrane). Co-localization of VAD1 with the different subcellular markers is shown in the merge panel (right). Confocal images were observed two days after agroinfiltration. Scale bars = 20μM. (B) Transient co-expression by agroinfiltration of *N*. *benthamiana* leaves of *35S*::*RFP*::*ΔVASt* construct (left panels), with subcellular markers (central panels, ERD2–GFP labels the Golgi, γ-TIP–GFP labels the tonoplast, GFP labels the cytoplasm, MYB30-GFP labels the nucleus, PMA4–GFP labels the plasma membrane (Plasma m.). Co-localization of VAD1 with the different subcellular markers is shown in the merge panel (right). Localization of *35S*::*RFP*::*ΔVASt* is not observed in the nucleus. Confocal images were observed two days after agroinfiltration. Bars = 20 μM.

To monitor the subcellular localization of the VAD1 protein, a C-terminal RFP fusion construct under the control of the 35S promoter was expressed in the leaf epidermal cells of *Nicotiana benthamiana* leaves by agroinfiltration. Using a Leica SP8 laser confocal microscope, VAD1 was observed in the Golgi apparatus, the cytoplasm, the nucleus, the tonoplast and the plasma membrane (Fig 2A, left panels). This multiple localization was confirmed by co-expression with different subcellular markers (Fig 2A, central panels) [11, 30, 38, 32, 33]. Similar observations were done by transient expression of the same constructs in *Arabidopsis thaliana* leaves [39] (S2A Fig). *35S*::*RFP*::*VAD1* was not observed in mitochondria, peroxysomes (data not shown) and chloroplasts (Fig 2A). These observations were also confirmed using a C-terminal GFP fusion construct under the control of the *VAD1* promoter (S2C Fig), with the exception of the nucleus where expression was not, or rarely, observed.

### The VASt domain of VAD1 is necessary for VAD1 nuclear localization and suppression of HR

**Fig 3:**
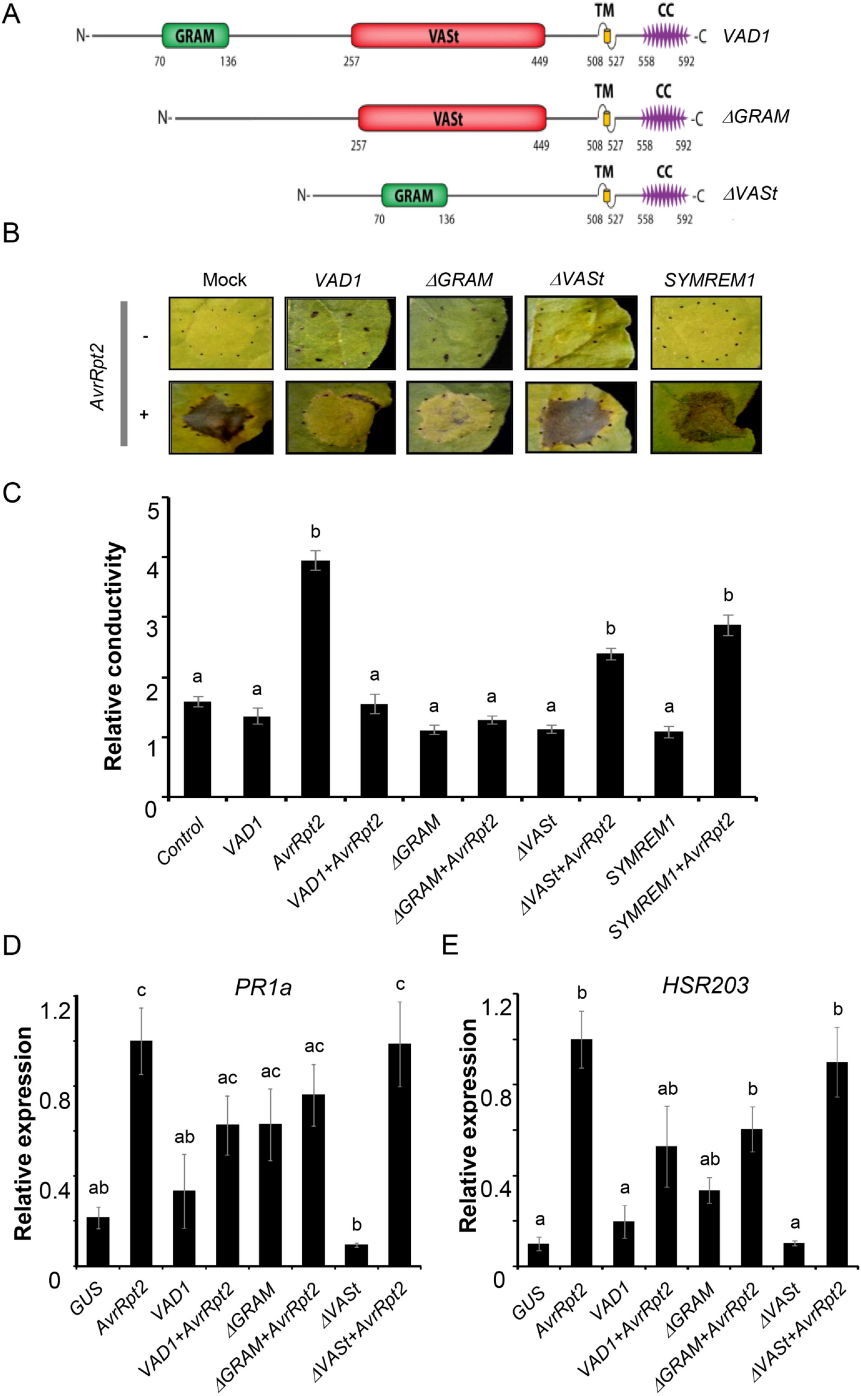
Structure-function analysis of *VAD1* effects on HR cell death and defense in response to bacterial avirulence effectors. (A) Schematic representation of VAD1 and constructs. GRAM (Glucosyltransferases, Rab-like GTPase Activators, Myotubularins) domain; VASt (VAD1 analog of START) domain; TM, transmembrane helix; CC: Coiled-coil. Residue number corresponds to amino acid position of VAD1 domains. (B) Observation of HR induced by AvrRpt2 after agroinfiltration of *N*. *benthamiana* leaves alone or co-expressed with the constructs *35S*::*VAD1*, *35S*::*ΔGRAM*::*VAD1*, *35S*::*ΔVASt*::*VAD1* or *35S*::*SYMREM1*. Observations were made 24h post-infiltration. (C) Quantification of cell death by measuring electrolyte leakage 24h after agroinfiltration of *N*. *benthamiana* leaves with the indicated strains (OD 0.3). Data are expressed relative to AvrRpt2 data at 1 h after sampling. Statistically significant differences were determined using Kruskal and Wallis one-way analysis of variance (letters indicate P < 0.05). (D and E) Expression analysis of the *PR1a* defense gene and *HSR203J* gene in *N*. *benthamiana* leaves after agroinfiltration with the indicated strains (OD 0.3) Data are presented as means ± SE (n ≥ 12), and error bars represent standard error. Letters indicate a significant difference between tested construct and control at P<0.05 by Kruskal and Wallis one-way analysis of variance test.

We previously reported that VAD1 harbors several conserved domains: a GRAM putative lipid binding domain at the N-terminal region (position 116–182), a VASt (VAD1 analog to START) domain (position 257–449), a transmembrane domain and a coiled-coil domain (Fig 3A)[5]. To determine if a particular VAD1 domain is necessary for its effects on HR and defense, deleted versions of VAD1 lacking either the GRAM domain or the VASt domain were used (Fig 3A). First we tested the effect of overexpression of these constructs by *Agrobacterium*-mediated transient expression in leaf epidermal cells of *N*. *benthamiana* leaves, on the HR induced by the expression of the *Pseudomonas* effector AvrRpt2, as described before. While the VAD1-*Δ*GRAM deletion did not significantly alter the negative effect of VAD1 on the development of the AvrRpt2-triggered HR, the VAD1-*Δ*VASt deletion strongly reduced this effect (Fig 3B). These visual observations were confirmed by ion leakage measurements in leaf disk assays 24h after plant agro-infiltration with the different strains (Fig 3C). *PR1a* and *HSR203J* (a well known marker of HR cell death) [40, 41] gene expression was also correlated with the visual observations, showing a similar level when AvrRpt2 alone, or AvrRpt2 + VAD1-ΔVASt were co-expressed (Fig 3D and 3E). Similar results were observed when we tested the effect of the same constructs on HR induced by another avirulence gene *AvrPto* [42], S1 Fig). Interestingly, the localization of VAD1 in multiple subcellular compartments was not affected by the GRAM or VASt domain deletions, excepted in one case: the nuclear localization of VAD1 was abolished by the *Δ*VASt deletion (Fig 2B and S2B Fig). Finally, expression of proteins fused to RFP was confirmed by Western blot in all cases (S3 Fig), showing that subcellular detection of RFP fluorescence in different compartments is due to localization of VAD1 and not passive diffusion of truncated RFP. In addition, while the proteins cannot be detected in presence of AvrRpt2, probably as a result of protein degradation [43, 44], they can be detected in the case of AvrPto, suggesting that even if they are present at low levels and/or transiently before degradation by AvrRpt2, they induce the observed effects on HR.

Taken together, these data show that (i) VAD1 mediated effects on HR cell death and defense expression is not dependent on the presence of the GRAM domain, (ii) the VASt domain is essential for VAD1 activity as an HR cell death repressor, and for its nuclear localization.

### *VAD1* overexpressing plants exhibit increased susceptibility to a virulent strain of the bacterial pathogen *Pseudomonas syringae*

**Fig 4:**
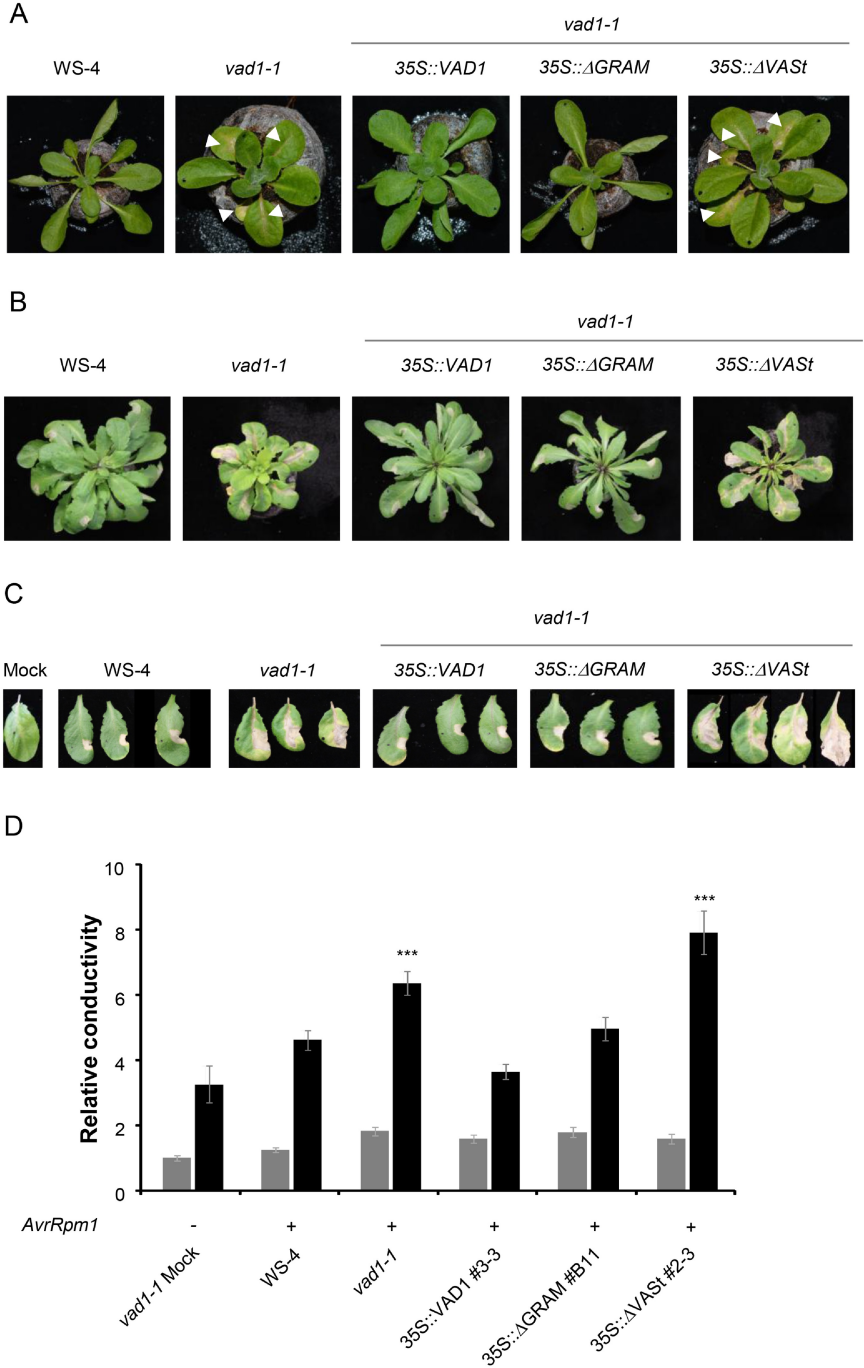
Overexpression of *VAD1*, but not *VAD1* lacking the VASt domain, leads to suppression of cell death and defense phenotypes in *vad1-1* during plant development and in response to bacterial avirulent pathogens. (A) Four-week old *vad1-1* plants and transgenic *vad1-1* plants containing the *35S*::*RFP*::*ΔVASt* construct show typical cell death lesions (marked by white arrows), while transgenic *vad1-1* plants containing the *35S*::*RFP*::*VAD1* construct or the *35S*::*RFP*::*ΔGRAM* construct show a wild type phenotype. (B-C) Five-week old plants have been inoculated with suspensions (2.10^6^ colony-forming units (CFU/mL) of *Pst* strain DC3000 expressing AvrRpm1. Wild type and transgenic *35S*::*RFP*::*VAD1* and *35S*::*RFP*::*ΔGRAM* plants showed typical HR lesions, while *vad1-1* and transgenic plants containing the construct *35S*::*RFP*::*ΔVASt* construct showed run away cell death. These phenotypes were observed 3 days post-inoculation (B) or 6 days post-inoculation (C). (D) Quantification of cell death by measuring electrolyte leakage 24 (grey bars) and 48h (black bars) after infiltration of leaves with *Pst* strain DC3000 AvrRpm1 (2x10^7^ colony-forming units (CFU/mL). Data are expressed relative to WS-4 1h after sampling. One transgenic line is shown per construct, out of 2–3 analyzed with similar results. Statistically significant differences were determined using Kruskal and Wallis one-way analysis of variance followed by nonparametric multiple comparison (* indicates P < 0.05).

To get a deeper understanding of the potential of VAD1 at the entire plant level, we produced transgenic Arabidopsis in the *vad1-1* mutant background expressing a green-fluorescing protein fusion of *VAD1* under control of the cauliflower mosaic virus CaMV35S promoter and the deleted versions of *VAD1* lacking either the GRAM domain or the VASt domain, already used (Fig 3A). Transgene expression was confirmed in all generations by western blots (S3C Fig). Transgenic *35S*::*VAD1* and *35S*::*ΔGRAM*::*VAD1* plants developed similarly to the wild type in T0 and following generations (Fig 4A). However a cell death phenotype similar to the one observed typically for *vad1-1*, was observed in the case of the transgenic *35S*::*ΔVASt*::*VAD1* (Fig 4A), confirming that the VASt domain is essential for VAD1 activity as an HR cell death repressor. In addition, plant development was affected, the transgenic *35S*::*ΔVASt*::*VAD1* plants showing a smaller rosette than the wild type and presenting *vad1-1* leaf morphology in relation to lesion appearance [14]. These data were quantitatively confirmed by measuring the percentage of leaves with lesions in the different lines between 17 and 31 days post-transplanting (Table 1). In response to inoculation with *Pst* DC3000 AvrRpm1, a typical HR was observed in the *vad1-1* plants complemented with *35S*::*VAD1* and *35S*::*ΔGRAM*::*VAD1* (Fig 4B and 4C). In contrast, in the mutant *vad1-1* and the transgenic *35S*::*ΔVASt*::*VAD1*, the leaves that display propagative lesions along the whole primary vein showed spreading cell death (runaway cell death, RCD), as previously shown for *vad1-1* [14](Fig 4B and 4C). These visual observations were confirmed by ion leakage measurements in leaf disk assays 24h after inoculation with *Pst* DC3000 *AvrRpm1* (Fig 4D).

**Table 1:** Percentage of leaves with lesions in the different plant lines, at several times (17, 21, 24 and 28 days post-transplanting) of plant development, under normal growth conditions.

Plant Lines	% Leaves with lesions			
	17	21	24	28
Ws-4	0	0	0	0
*vad1-1*	0	7	20	70
*vad1-1/35S*::*VAD1*	0	0	0	0
*vad1-1/ 35S*::*ΔGRAM*	0	0	0	0
*vad1-1/ 35S*:: *ΔVASt#4–1*	0	7	17	46
*vad1-1/ 35S*::*ΔVASt#2–3*	0	15	19	63

One representative experiment out of three experiments is shown. 12–21 plants per line have been used in the experiment.

**Fig 5:**
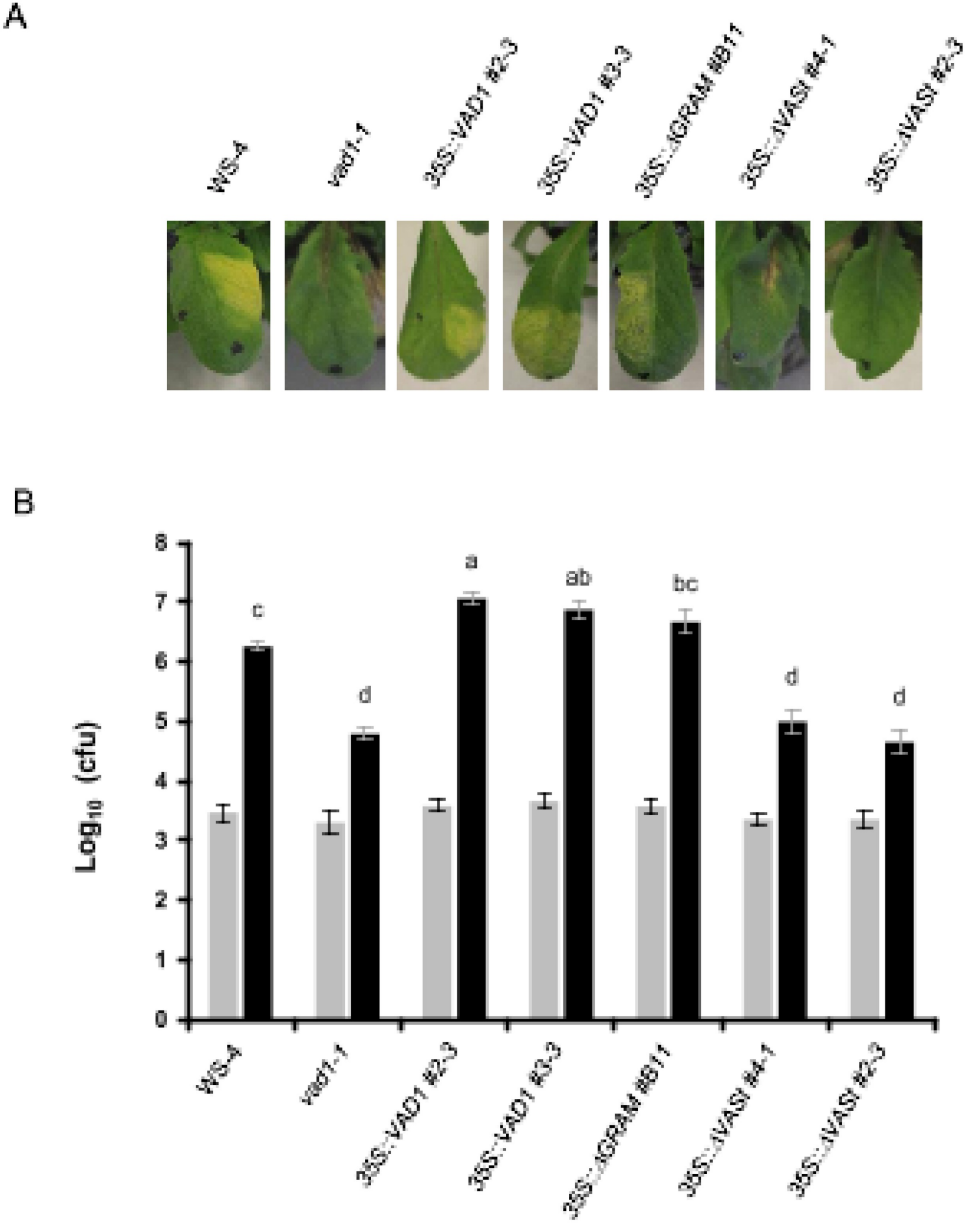
Overexpression of *VAD1*, but not *VAD1* lacking the VASt domain, leads to enhanced susceptibility in *vad1-1* in response to the virulent strain *Pst* DC3000. (A) Four-week old wild type and transgenic *vad1-1* plants containing the *35S*::*RFP*::*VAD1* construct or the *35S*::*RFP*::*ΔGRAM* construct show a typical chlorosis 72h after inoculation with suspensions (5x10^6^ colony-forming units (CFU/mL) of *Pst* strain DC3000. *vad1-1* plants and transgenic *vad1-1* plants containing the *35S*::*RFP*::*ΔVASt* construct do not show (or very faint) symptoms. (B) Growth of *Pst* DC3000 in the different lines indicated. Leaves of four-week old plants were inoculated with a bacterial suspension (2x10^5^ CFU/mL) of *Pst* strain DC3000 and bacterial growth was measured 0 (grey bars) and 3 (black bars) days after inoculation. Data were collected from four independent experiments with five individual plants (four leaves/plant) per point. Statistical differences using Kruskal and Wallis one-way analysis of variance followed by the Conover's-test for multiple comparisons analysis of variance (P value < 0.05) are indicated by letters.

We next examined the response of the same transgenic lines to a virulent strain of *Pst* (strain DC3000)(Fig 5A). A typical chlorosis was observed in the case of the wild type and the transgenic *vad1-1* plants containing the *35S*::*RFP*::*VAD1* construct or the *35S*::*RFP*::*ΔGRAM* construct, while very faint or no symptoms were observed in the mutant *vad1-1*, as previously reported [14]. The same weak phenotype was observed for the transgenic *vad1-1* plants containing the *35S*::*RFP*::*ΔVASt* construct. These observations were confirmed by evaluation of *in planta* bacterial growth (Fig 5B). No significant differences in resistance could be found between the *vad1-1* mutant and the two transgenic lines expressing the *35S*::*RFP*::*ΔVASt* construct, indicating that this construct is unable to complement the mutant phenotype. Interestingly, in the transgenic lines expressing the *35S*::*RFP*::*VAD1* construct or the *35S*::*RFP*::*ΔGRAM* construct, a significantly increased susceptibility was observed as compared to the wild type. Therefore, overexpression of *VAD1* leads to increased susceptibility to virulent strains of *Pst*.

## Discussion

The mechanistic regulation of PCD is a fundamental question in plant biology. Our understanding of this biological process in response to environmental stresses including pathogens, came for a large part from the identification of mutants displaying spontaneous HR-like cell death, the LMMs [1, 10]. Indeed, a considerable number of reports link these LMM phenotypes to ETI components (*R* genes and signaling pathways)[16]. However, the functions of these negative regulators of immunity and cell death remain poorly understood.

Here we demonstrate that overexpression of *VAD1* controls negatively the HR cell death and defense expression either transiently in *N*. *benthamania* or in Arabidopsis plants in response to avirulent strains of *Pseudomonas syringae*. Bax-Inhibitor-1 (BI-1), a conserved cell death regulator in yeast, animals and plants, was also shown to suppress elicitor-induced cell death [45]. Importantly, its overexpression conferred reduced susceptibility to the necrotrophic pathogen *Fusarium graminearum* and decreased resistance to the biotrophic parasite *Blumeria graminis* f.sp. *hordei*, suggesting that it can modulate cell death in interaction with plant pathogens of different lifestyles [46]. Interestingly, another protein, structurally related to VAD1 and which has been recently identified [47], is required for regulation of cell death and basal resistance to *Botrytis cinerea*. This C2 GRAM domain-containing protein is an interactor of the Arabidopsis BAG6 protein (BAGP1, for BAG-Associated GRAM Protein). BAGP1 is required for BAG6 processing and this cleavage is required for resistance to *Botrytis*. BAGP1 is one of the rare examples among the numerous and ubiquitous C2 GRAM domain containing proteins, for which a function is known: regulation of autophagic cell death and resistance to *Botrytis*, functions similar to those identified to VAD1. In this context, our data support that over-expression of *VAD1* weakens defense and resistance to the biotrophic bacterial pathogen *Pst* DC3000 (Fig 5). This is in good agreement with the autoimmune phenotype observed in the mutant. This is also in line with *VAD1* gene expression pattern during plant–pathogen interactions [14]. Indeed, *VAD1* is triggered early in response to inoculation with avirulent strains of *Xanthomonas* and *Pseudomonas*, and then rapidly repressed to reach the background level observed in healthy plants. Altogether these results demonstrate that VAD1 controls HR cell death kinetics and intensity and defense. However, VAD1 is involved in the control of cell death propagation and not its initiation, in contrast to most *LMM* genes characterized to date [14]. Our observations (Fig 4) of the run-away cell death phenotype in the mutant line in one hand, and of highly limited HR cell death in the overexpressor line, are clearly in favor of this hypothesis.

Further insights into the roles of VAD1 in the control of plant defense and cell death control should be gained from examination of its biochemical function. VAD1 encodes a protein harboring a GRAM domain predicted to bind lipids [14] and a VASt domain related to Bet v1-like domains known to bind hydrophobic ligands such as phytohormones, lipids and polyketides [5]. In the present study, molecular dissection of the *VAD1* gene demonstrated that the VASt domain is necessary for the inhibition of pathogen-induced cell death in *N*. *benthamiana* and in *Arabidopsis thaliana*. *Δ*VASt-VAD1 (lacking the internal 181 amino acid residues of the VASt domain of VAD1) completely abolished the cell death–suppression ability. On the other hand, *Δ*GRAM-VAD1, in which the N-terminal 65 amino acids of VAD1 were deleted, maintained its activity. Considering that VASt and GRAM domains are (i) associated in nearly all the eukaryotic VASt containing proteins, (ii) are both supposed to be involved in lipid/hydrophobic ligand [5], it is rather surprising to find that only the VASt domain is essential for cell death suppressor activity. In this context, VAD1 is clearly different from the mammalian CERT protein, with which VAD1 shares a common domain structure involving a PH superfamily domain (PH and GRAM respectively) and a Bet v1-like superfamily domain (START and VASt respectively)[5]. Cooperation between the PH and START domains in CERT is critical for its function as a ceramide transport protein [48]. This suggests that the two VAD1 domains (GRAM and VASt) are probably involved in different functions and/or behave independently.

Another interesting feature of the VASt domain is related to the subcellular localization of VAD1. Analysis of the plant cellular localization of VAD-1 indicated that the localization of VAD1 was in multiple subcellular compartments and was not affected by the GRAM or VASt domain deletions, excepted in one case: the nuclear localization of VAD1 was abolished by the *Δ*VASt deletion (Fig 2). While deletion of the VASt domain leads to abolition of both nuclear localization and cell death suppressor activity, it is tempting to hypothesize that the VASt domain is crucial for cell death activity through its nuclear localization. However, the fact that when the analysis of the VAD-1 cellular localization was performed with a C-terminal GFP fusion construct under the control of the *VAD1* promoter (instead of a construct 35S::RFP::VAD1), a much lower level of expression was observed in all compartments, especially in the nucleus (almost undetectable), makes this hypothesis difficult to verify. Other approaches would be necessary to clarify this point. Subcellular localization of plant cell death regulator proteins has been investigated only in a few cases. For example, the C-terminal region of BnBI-1 is located on the cytosolic face of the ER [49], and supposed to play an important role in cell death regulation, through a potential role at the level of ER membrane homeostasis in relation to oxidative stress. However, the precise mechanism of cell death suppression by BI-1 remains unknown. Another Arabidopsis cell death regulator, *EDR2*, encodes a protein with a structural organization close to that of VAD1 and consisting of a PH domain, START domain and a plant-specific (DUF1336) domain of unknown function [50, 51]. EDR2 localizes to the plasma membrane, endosomes and endoplasmic reticulum [51] and its PH domain (close to GRAM domain) has been shown to bind specifically to phosphatidylinositol- 4-phosphate *in vitro*. However, the function of the START or the DUF1336 domains has not been characterized, either biochemically or in relation to subcellular protein localization. Finally, among numerous proteins containing both a GRAM and a VASt domain, Ysp2p, one of six StART-like proteins in yeast, has two StART-like domains which have been recently demonstrated both to bind sterol [20]. Interestingly, localization of YSP2 in the ER at contact sites with the plasma membrane, has been shown to be essential for its function, potentially involved in the rate of transfer of sterol from PM to ER [52]. However, the specific role of the START domains has not been addressed genetically. To our knowledge, the present work constitutes the first structure-function analysis performed on such cell death related proteins containing both a GRAM and a VASt domain. Although the exact function of the VASt domain of VAD1 remains to be discovered, its essential role in VAD1 cell death regulation has been genetically demonstrated.

Now, an important step in unraveling the role of VAD1 in restricting cell death in plant-pathogen interactions, would be to determine the ligand(s) (lipid, hormone or other molecules) bound by the VASt domain, which has been demonstrated here to be essential for the cell death regulatory function of VAD1. Another powerful, but more indirect approach, to understand VAD1-mediated cell death and defense responses, would be to identify additional components in the VAD1 signaling pathway, by conducting a *vad1* suppressor screen.

## Supporting information

S1 Fig *VAD1* overexpression leads to reduction of HR cell death and defense in response to bacterial avirulence effectors. Observation of HR induced by AvrPto after agroinfiltration of *N*. *benthamiana* leaves alone or co-expressed with the constructs *35S*::*RFP*::*VAD1*, *35S*::*RFP*::*ΔGRAM*, *35S*::*RFP*::*ΔVASt*. Observations were made 56h post-inoculation. (B) Quantification of cell death by measuring electrolyte leakage 48h after agroinfiltration of *N*. *benthamiana* leaves with the indicated strains (OD 0.5). Data are expressed relative to AvrPto data at 1h after sampling. Statistically significant differences were determined using Kruskal and Wallis one-way analysis of variance followed by nonparametric multiple comparison (* indicates P < 0.05).
(TIF)

S2 Fig VAD1 localizes in multiple subcellular compartments and a VASt domain deletion excludes VAD1 from the nucleus in *Arabidopsis thaliana*. Transient co-expression by agroinfiltration of *Arabidopsis thaliana* leaves of *35S*::*RFP*::*VAD1* construct (A), 35S::RFP::*Δ*VASt construct (B) or pVAD::VAD1::GFP (C), the construct alone (left panels), with subcellular markers (central panels, ERD2–GFP labels the golgi/endoplasmic reticulum, MYB30-GFP labels the nucleus). Co-localization of VAD1 with the different subcellular markers is shown in the merge panel (right). Confocal images were observed two days after agroinfiltration. Scale bars = 20 μM.
(TIF)

S3 Fig VAD1 protein detection in protein extracts from *Nicotiana* leaves after transient expression or in Arabidopsis transgenic lines. Transient expression of VAD1 in presence or not of bacterial avirulence effectors: *N*. *benthamiana* leaves were agroinfiltrated with the constructs *35S*::*RFP*::*VAD1*, *35S*::*RFP*::*ΔGRAM*, *35S*::*ΔVASt*::*VAD1*, *35S*::*SYMREM1* alone, or co-expressed with (A) AvrRpt2 (OD 0.3) or (B) AvrPto (OD 0.5). Leaves were harvested 24h post-inoculation. Blots of total leaf protein extracts were incubated with RFP antibody or GFP antibody. The full-length RFP-VAD1, SymREM1-CFP protein or truncated VAD1 proteins VAD1-*Δ*GRAM and VAD1-*Δ*VAST fused to the RFP are predicted to be respectively 94 kDa, 60 kDa, 86 kDa and 73 kDa. Western blot shows VAD1 protein detection (VAD) and VAD1 derivatives (*Δ*GRAM, *Δ*VASt). VAD1 and derivatives are not detectable in the presence of AvrRpt2. Ponceau S staining confirms equal loading. These experiments were repeated three times with similar results. (C) Immunoblot analysis of total protein extracts from 2–3 week old T3 transgenic plants, separated by SDS-PAGE and probed with α-RFP antibody. Transgenic lines were generated in the WS-4 *vad1* background. Ponceau S staining of the membrane confirms equal loading. Arrows on the left indicate protein positions.
(EPS)

S1 Table Primers used in this work. (DOCX)
