# Supplementary figures and images for: An essential role for the VASt domain of the Arabidopsis VAD1 protein in the regulation of defense and cell death in response to pathogens
Source: PLoS One. 2017 Jul 6;12(7):e0179782. doi: 10.1371/journal.pone.0179782 (PMC5500287; doi:10.1371/journal.pone.0179782)

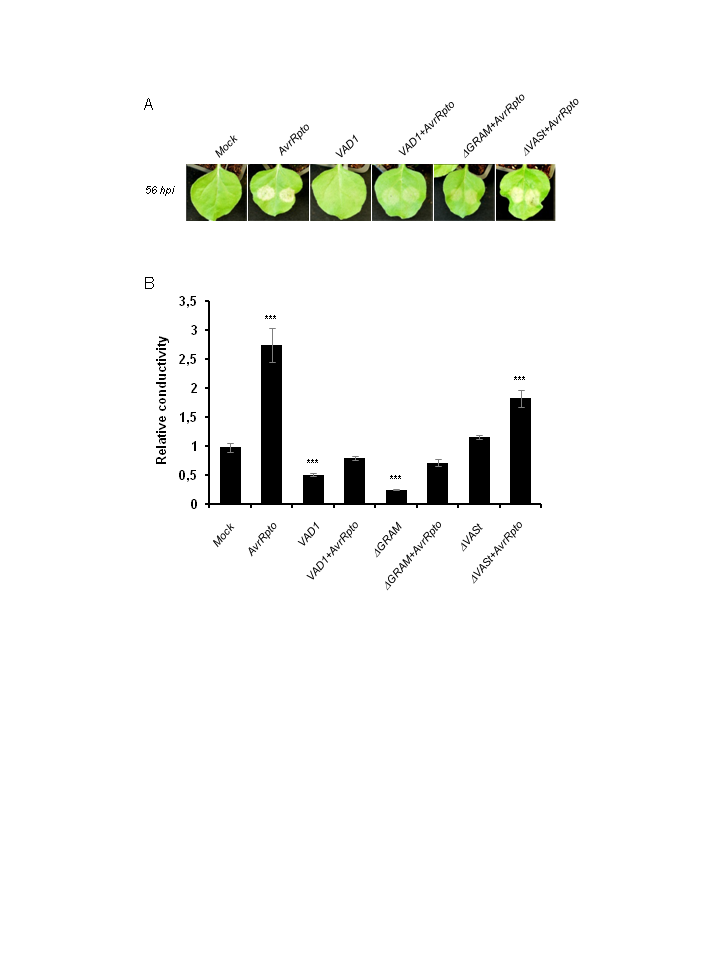

Supplement: S1 Fig — Observation of HR induced by AvrPto after agroinfiltration of N. benthamiana leaves alone or co-expressed with the constructs 35S::RFP::VAD1, 35S::RFP::ΔGRAM, 35S::RFP::ΔVASt. Observations were made 56h post-inoculation. (B) Quantification of cell death by measuring electrolyte leakage 48h after agroinfiltration of N. benthamiana leaves with the indicated strains (OD 0.5). Data are expressed relative to AvrPto data at 1h after sampling. Statistically significant differences were determined using Kruskal and Wallis one-way analysis of variance followed by nonparametric multiple comparison (* indicates P < 0.05). (TIF) [file pone.0179782.s001.tif]

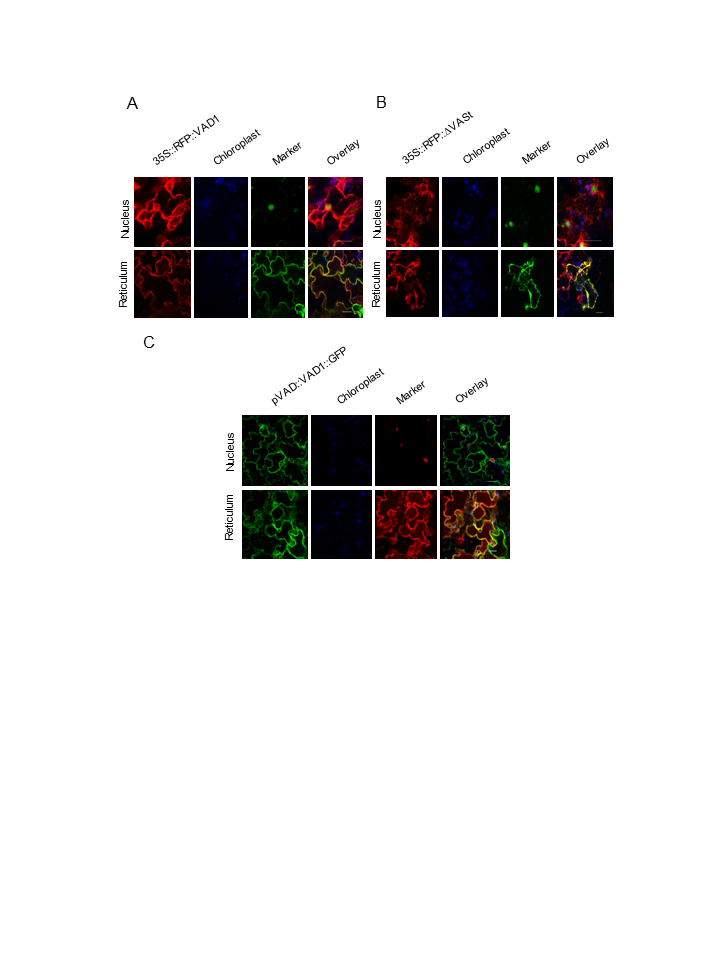

Supplement: S2 Fig — Transient co-expression by agroinfiltration of Arabidopsis thaliana leaves of 35S::RFP::VAD1 construct (A), 35S::RFP::ΔVASt construct (B) or pVAD::VAD1::GFP (C), the construct alone (left panels), with subcellular markers (central panels, ERD2–GFP labels the golgi/endoplasmic reticulum, MYB30-GFP labels the nucleus). Co-localization of VAD1 with the different subcellular markers is shown in the merge panel (right). Confocal images were observed two days after agroinfiltration. Scale bars = 20 μM. (TIF) [file pone.0179782.s002.TIF]
