# Supplementary material for: An essential role for the VASt domain of the Arabidopsis VAD1 protein in the regulation of defense and cell death in response to pathogens
Source: PLoS One. 2017 Jul 6;12(7):e0179782. doi: 10.1371/journal.pone.0179782 (PMC5500287; doi:10.1371/journal.pone.0179782)
Supplement: S1 Table — (DOCX) [file pone.0179782.s004.docx]

Table S1 Primers used in this study

| Name | Forward | Reverse |
| --- | --- | --- |
| VAD1 | GGGGACAAGTTTGTACAAAAAAGCAGGCTTCATGGCGATGCTTTCTACTGCTTC | GGGGACCACTTTGTACAAGAAAGCTGGGTTCTTACTGTTTGTTGCGGCGGA |
| ∆VASt | CAACTGGAAGCCTGAAGATACAGATGCACCTAAAGGGAATAAGTTGATTGAGGATGGTGAAC | CTGCTAGCGGTTCACCATCCTCAATCAACTTATTCCCTTTAGGTGCATCTGTATCTTCAGGC |
| ∆GRAM_BamHI | CGGGATCCATGGGCCAAGAAAGTGAGAG |  |
| ∆GRAM_KpnI |  | GGAGACTTCAGTATAGGTTCTCAC |
| ΔGRAM_stop |  | GAGGCGGCGTTGTTTGTCATT |
| F45030BamHI | CG**GGATCC**GTATGTGTCTCTAACCACC |  |
| RendNot |  | AAGGAAAAAAGCGGCCGCCTGTTTGTTGCGGCGGAG |
| F-Box | GGCACTCACAAACGTCTATTTC | ACCTGGGAGGCATCCTGCTTAT |
| HSR203J | ATGGTTCATGAAAAGCAAGTG | GCGTGTAGACAGTGTAGTAC |
| PR1a | CCTTTATGTACGTGTGTATGC | GTAGGTGCTCTTGTTCTTCCC |
